# A Landmark Case of Childhood Glaucoma Care in Bangladesh: Gonioscopy-Assisted Transluminal Trabeculotomy in Primary Congenital Glaucoma

**DOI:** 10.7759/cureus.79492

**Published:** 2025-02-23

**Authors:** Md Iftekher Iqbal, Fariah Osman

**Affiliations:** 1 Department of Ophthalmology, Bangladesh Eye Hospital, Dhaka, BGD

**Keywords:** bangladesh, childhood glaucoma, gatt, primary congenital glaucoma, trabeculotomy

## Abstract

Primary congenital glaucoma (PCG) is a challenging condition requiring early surgical intervention to prevent visual impairment. Gonioscopy-assisted transluminal trabeculotomy (GATT) is a novel minimally invasive procedure that presents a promising alternative for the management of childhood glaucoma, utilizing either a microcatheter or suture (e.g., 5-0 prolene). We present a case of a three-year-old male from consanguineous parents diagnosed with bilateral PCG, who underwent suture-GATT with 5-0 prolene in his left eye. A six-month postoperative follow-up showed no complications and good control of intraocular pressure (IOP) following the procedure. By showing good IOP control over a mid-term follow-up in a Bangladeshi patient, this case demonstrates the efficacy of suture-GATT in treating pediatric glaucoma.

## Introduction

High intraocular pressure (IOP) is a hallmark of many childhood glaucoma-related conditions. If these conditions are not treated, they can lead to glaucomatous optic neuropathy and vision loss. Glaucoma, impacting over 300,000 children globally, accounts for 5% of blindness within the pediatric population [1].

Primary congenital glaucoma (PCG) is classified as one of the subtypes of childhood glaucoma according to the Childhood Glaucoma Research Network (CGRN) classification system [2]. It is recognized as the most prevalent type, with an incidence rate of approximately one in 10,000 to 20,000 live births in the Western population, while in India, the incidence can be as high as one in 3,300 live births [3]. This hereditary condition affects the trabecular meshwork (TM) and the anterior chamber angle, resulting in elevated IOP [4]. Children diagnosed with PCG often exhibit buphthalmos, corneal edema accompanied by haziness, and ruptures of Descemet's membrane (Haab's striae) [5].

Surgical intervention, specifically ab-externo and ab-interno trabecular incisional surgeries (trabeculotomy), is the primary treatment for PCG [6]. Trabeculotomy lowers the pressure inside the eye by improving the flow of aqueous humor through Schlemm's canal and the collector channels around it without a bleb formation [7,8].

In the late 1960s, trabeculotomy ab-externo was introduced to treat PCG and juvenile open-angle glaucoma (JOAG) [9,10]. Over the years, the trabeculotomy technique has changed from using a trabectome to a suture trabeculotomy to manage IOP in children with glaucoma [8].

The advancement of minimally invasive glaucoma surgery (MIGS) has transformed treatment protocols for glaucoma, with gonioscopy-assisted transluminal trabeculotomy (GATT) demonstrating comparable efficacy to trabeculotomy ab-externo in patients with PCG. Modern advances include Schlemm's canal microcatheterization and transluminal viscodilation followed by ab-interno trabeculotomy with the OMNI surgical system (Sight Sciences, Menlo Park, CA, USA) [11-13].

In this report, we present a case of a three-year-old boy with PCG who underwent a successful suture-GATT with 5-0 prolene. To our knowledge, this is the first documented case of GATT from Bangladesh, and the authors believe it will contribute to future work on pediatric glaucoma care in Bangladesh.

## Case presentation

The study adhered to the principles outlined in the Declaration of Helsinki and received approval from the institutional review board of the affiliated hospital. The patient's parents provided informed written consent for the surgical procedure and the publication of this study.

A three-year-old boy from consanguineous parents was referred by a pediatrician with a three-month history of enlarging eyeballs, a hazy right cornea, and intermittent watering from both eyes.

The parents, hailing from a low socioeconomic background, noted that this child is their third, delivered vaginally at full term. Alarmingly, both of his siblings similarly faced serious eye problems since birth. His 17-year-old brother is bilaterally blind, while his 12-year-old brother has a history of ocular surgery in both eyes, though the parents were unable to provide detailed information regarding these procedures.

The gross visual acuity on clinical examination demonstrated fixation and followed an object in both eyes (OU) and bilateral buphthalmos.

**Figure 1:**
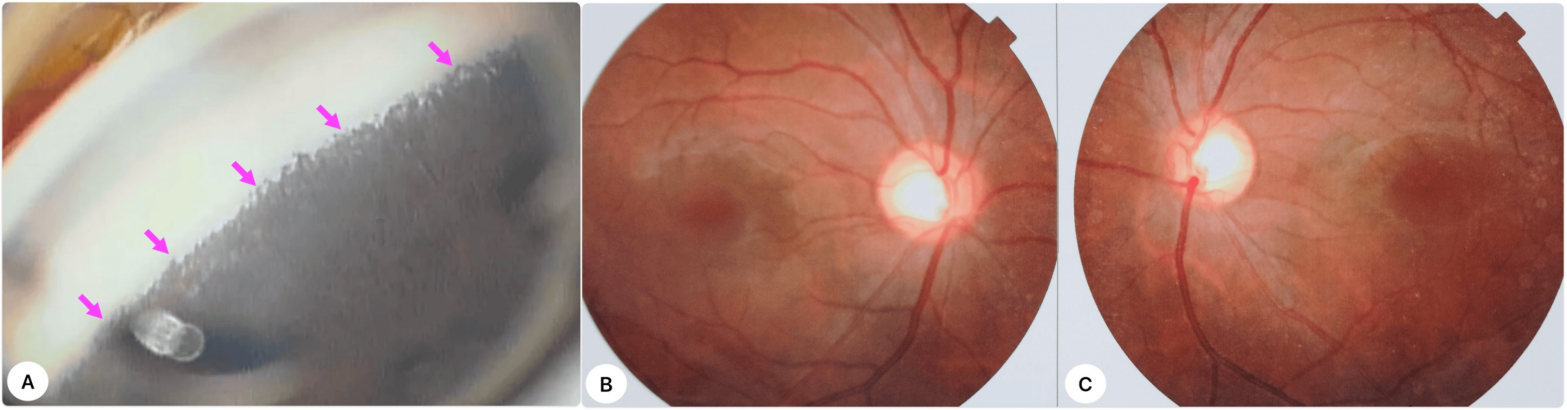
Examination findings A: anterior iris insertion (magenta arrows) on gonioscopy; B, C: fundus images of the right and left eye, respectively.

An examination under anesthesia (EUA) revealed significant corneal white-to-white (WTW) diameters of 14.5 mm in the right eye (OD) and 14 mm in the left eye (OS). While the OS displayed a clear cornea, the OD showed Haab's striae and notable bilateral deep, regular central, and peripheral anterior chamber depths. Intraocular pressure (IOP) measurements with an iCare tonometer (iCare USA, Raleigh, NC) showed 34 mmHg in OD and 28 mmHg in OS. A gonioscopic exam showed (Figure 1A) that the iris was inserted anteriorly on both eyes, and the trabecular meshwork (TM) and Schwalbe's line (SL) were not clearly defined. Direct ophthalmoscopic examination indicated deep cupping in both eyes, with a cup-to-disc ratio of 0.6 in OD (Figure 1B) and 0.7 in OS (Figure 1C), and no notable retinal pathology was observed. The axial length (AL) was measured at 22.45 mm for OD and 21.75 mm for OS. Objective refraction results were (-3.00 DS × -1.00 DC @ 180°) in OD and -2.75 DS in OS.

The patient received a diagnosis of PCG OU and was scheduled for mitomycin-C (MMC)-augmented trabeculectomy in OD and suture-GATT in OS. In the meantime, they also prescribed a combination of timolol 0.5% and brinzolamide 1%.

Surgical procedure

This patient underwent a suture-GATT procedure in the OS by one of the authors (MI I) at Bangladesh Eye Hospital and Institute, Dhaka, Bangladesh.

The surgeon made a tangential corneal paracentesis in the inferonasal quadrant to guide the thermally blunted 5-0 prolene suture. Next, the surgeon made a temporal paracentesis. The patient's head was rotated approximately 30° away from the surgeon to achieve optimal visualization of the nasal angle, and the microscope was tilted approximately 45°.

**Figure 2:**
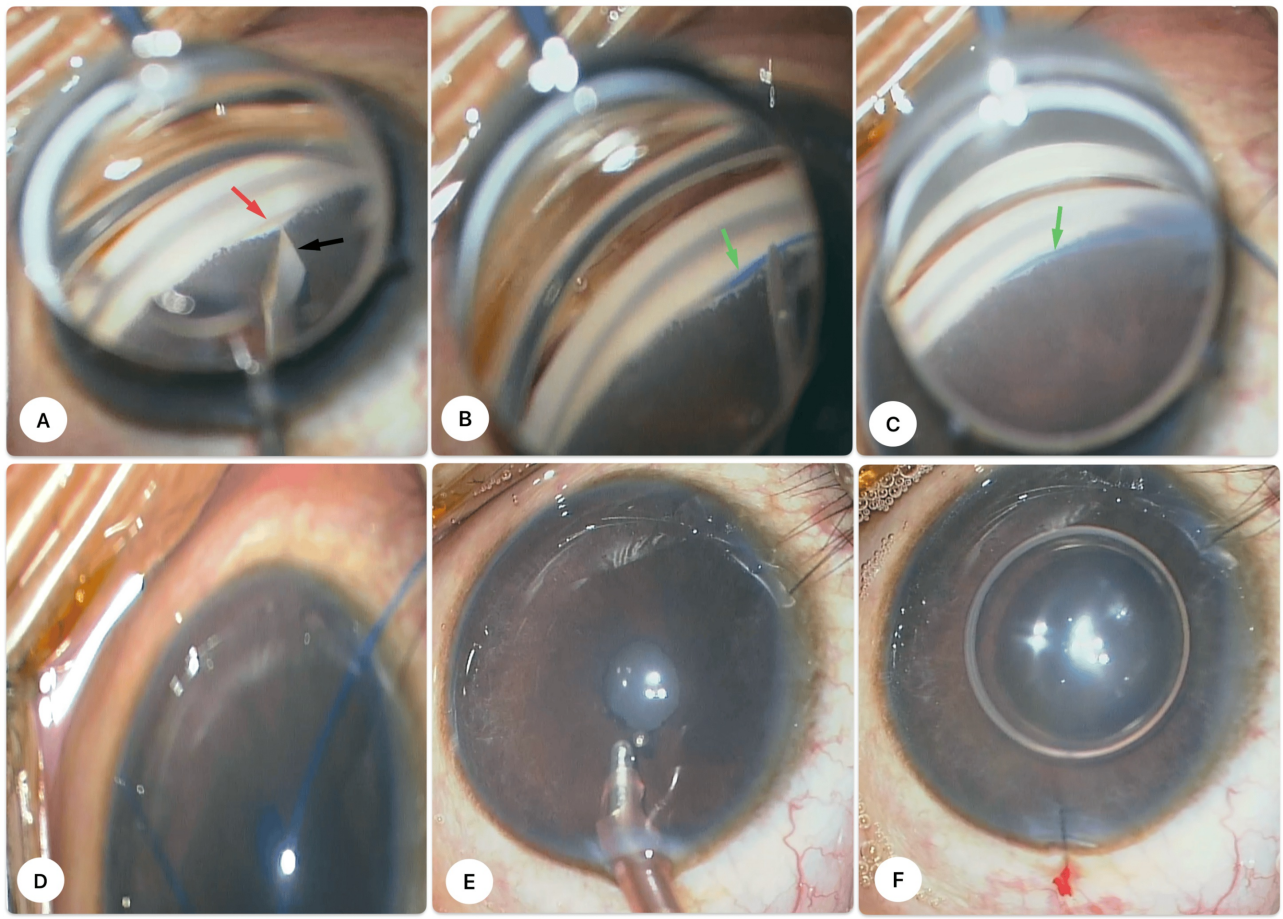
Intraoperative images demonstrating the key segments of the GATT in the left eye A: initial goniotomy (red arrow) with microvitreoretinal (MVR) blade (black arrow) to open up Schlemm’s canal; B, C: cannulation of Schlemm’s canal with a 5-0 prolene (green arrow) using a microsurgical forcep; D: retrieval of the 5-0 prolene within the anterior chamber using a microsurgical forcep after traveling around 180° circumference of the angle; E: aspiration of viscoelastic and hyphaema; F: well-formed, air-filled anterior chamber with corneal suture at temporal paracentesis. GATT: gonioscopy-assisted transluminal trabeculotomy

At the time of surgery, the anterior chamber was deepened with viscoelastic. With the Volk surgical gonio lens (Volk Optical, Inc., Ohio, USA), the iris was observed to cover the entire angle up to Schwalbe's line (Figure 2A), and angle structures were not visible. After gentle stripping of the iris from the angle with a blunt Sinsky hook, GATT was done without any intraoperative complications, and an electrocoagulation-induced thermally blunt-tipped 5-0 prolene was used. Initially, the surgeon performed a small goniotomy in the nasal TM using a microvitreortinal (MVR) blade through the temporal site (Figure 2A). Later, it involved inserting a 5-0 prolene into Schlemm's canal (SC) (Figure 2B) and circumferentially passing it around 180° of the canal (Figure 2C). As the prolene reached the 180° angle, it became stuck. It took several tries to get it through, but it was finally pulled out of the anterior chamber using microsurgical forceps (Figure 2D) to keep it from going into a false passage around the angle. So, hemi-GATT was ensured at that point. The viscoelastic and hyphema were aspirated (Figure 2E), and the anterior chamber was reformed with a balanced salt solution (BSS) and an air tamponade. The surgeon then used 10-0 nylon to seal the main paracentesis and BSS to make sure it was watertight (Figure 2F). To finish the procedure, 0.5 mL of intracameral moxifloxacin (0.8 mL) was injected.

Postoperative care

After removal of the eye patch two hours postoperatively, the patient was advised to instill topical moxifloxacin (0.5%) six times daily for a month, prednisolone acetate (1%) hourly for a day then tapered over a month, pilocarpine (0.5%) twice a day for seven days, and continue brinzolamide 1%.

Postoperative follow-up

On the first postoperative day (POD), seven POD, thirty POD, and every follow-up day for the next six months, the IOP consistently dropped by about 12-14 mmHg in OS with brinzolamide (1%).

**Figure 3:**
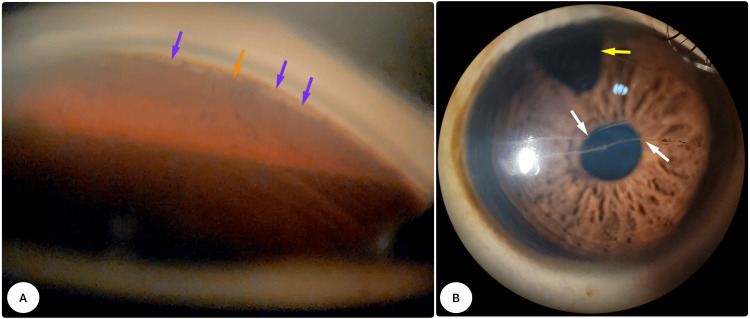
Postoperative status A: six-month postoperative goniograph of the left eye, cleaved trabecular shelf (purple arrows), and peripheral anterior synechiae (orange arrow); B: surgical peripheral iridectomy (yellow arrow) and Haab Striae (white arrows) in the right eye.

Figure 3A displays the gonioscopic image of the OS six months after surgery. It shows the open Schelmm's canal and the localized cleft, along with a few small areas of peripheral anterior synechiae and cleft closure.

The right eye had augmented trabeculectomy with 0.02% mitomycin-C (MMC) (Figure 3B). It has kept its good vision with a well-controlled IOP of about 10 mmHg over the last six months without needing any extra glaucoma medicines.

Both eyes were still having clinically stable glaucomatous changes in the optic nerve head until the last follow-up.

In the meantime, we referred the patient for vitreoretinal evaluation and vision rehabilitation with the pediatric ophthalmology department for further management.

## Discussion

According to the consensus of the World Glaucoma Association (WGA), trabeculotomy is regarded as the ideal intervention for PCG [5]. The most common strategy for circumferential trabeculotomy is the ab-externo technique, which necessitates a meticulous dissection of the conjunctival and scleral flaps and may adversely affect the success rate of subsequent trabeculectomy. These ab-externo methods are beneficial when the corneal clarity is impaired [11].

The gonioscopy-assisted transluminal trabeculotomy (GATT) technique was described by Grover et al. as a new approach to ab-interno circumferential trabeculotomy that spares the conjunctiva during open-angle glaucoma surgery [8].

The surgeons' training, preference, and ability to visualize angle structures determine whether they perform an ab-interno or ab-externo procedure [6]. We assert that our lead surgeon (MI I) possesses sufficient expertise in gonioscopy and angle-based surgical techniques to manage cases of PCG effectively [14,15].

Identifying the TM is still tricky, even with a clearer view during gonioscopy. This is because children’s TM has low pigmentation; the iris is attached anteriorly, there is translucent uveal tissue, and there is an absence of blood reflux into the Schlemm’s canal after lowering IOP during surgery [11]. Preoperative and intraoperative gonioscopy in our patient also demonstrated anterior iris insertion (Figure 1A), which made the angle structures barely visible initially. We used a blunt Sinsky hook to remove the overlying iris from the angle before performing the suture-GATT.

In the case of GATT, using a microcatheter equipped with a blinking head would ensure precise positioning within the angle [12,13]. However, it is expensive and impractical in a developing country like Bangladesh. In our study, we employed thermally blunted-tipped 5-0 prolene as outlined in various scholarly articles rather than utilizing a microcatheter with an indicator [5,3].

Several studies have shown that a microcatheter cannot turn 360° in one direction and usually stops at about 180° to 270° [16,17]. In this scenario, a microsurgical blade creates a small goniotomy and then removes the distal end. A surgeon can then pass a microcatheter in the opposite direction through an additional 23-gauge needle incision, thereby completing a 360° trabeculotomy. During the procedure, we encountered a situation in which the 5-0 prolene suture ceased movement after executing approximately a 180° turn. This occurrence may be indicative of significant angle dysgenesis, such as congenital stenosis or transection of the Schlemm's canal. The initial IOP was 28 mmHg, and there was moderate glaucomatous optic nerve damage in the left eye. We decided that a hemi-GATT procedure would be the best way to control the eye pressure, possibly along with using brinzolamide 1% if needed.

Postoperative complications associated with GATT frequently include iridodialysis, an increase in IOP, and hyphema, all of which require careful consideration [16]. These complications are likely attributable to the dissection of Schlemm’s canal, resulting in a communication between the anterior chamber and the suprachoroidal space [17]. Early IOP spikes may be caused by retained viscoelastic material, blood clots, or resolution of ciliochoroidal detachment, while delayed IOP spikes may be due to steroid response [5]. We experienced mild hyphema during the procedure, which ultimately resolved after a week, and over the six-month postoperative follow-up, his IOP in OS remained well-controlled with brinzolamide 1%.

Due to the elastic feature of a pediatric cornea, it is advisable to put a suture through the corneal wound [8]. We closed the main corneal wound with a 10-0 nylon suture to prevent wound leakage.

Trabeculectomy and tube shunts can cause serious complications, like hypotony, and long-term risks that may harm vision, including issues with corneal decompensation and bleb-related endophthalmitis. These are concerning, especially for those with prolonged life expectancy [11]. Since Haab's striae were present in the right eye (Figure 3B), it was difficult to view the angle with the goniolens. Therefore, we did an augmented trabeculectomy using 0.02% mitomycin-C (MMC). The surgery has successfully maintained good intraocular pressure (IOP) without the need for antiglaucoma medications for about six months following the procedure. We advised the parents about the potential risks that may arise and emphasized the importance of seeking immediate consultation if any concerns develop.

One of our study’s strengths is the recently developed ab-interno procedure, GATT, which not only maintained comparable IOP control but also reduced ab-externo-related complications, like scarring, leakage, or endophthalmitis [5,8].

The limitations of our study include a comparison with 360° trabeculotomy, as a more significant reduction in IOP is correlated with a higher extent of angle surgery, as well as a short follow-up duration of six months [8,11]. During the latest check-up for our patient, he had a gonioscopic exam six months after his surgery. The exam showed an open trabecular shelf and some peripheral anterior synechiae (Figure 3A). This "open shelf" indicates an open collector channel, typically linked to favorable postoperative IOP control [8].

The prognosis associated with PCG depends upon several factors, including the timing of initial presentation, the promptness of surgical intervention, the extent of optic nerve damage, the characteristics and quality of corneal enlargement and astigmatism, the progression of refractive error, and the presence of anisometropic amblyopia. Measuring how well children see and how much their vision has declined is difficult, making these methods less useful for tracking patients than measuring corneal diameter and eye pressure (IOP). So, it is essential to engage with a vitreoretinal specialist and a pediatric ophthalmologist and consider visual rehabilitation, both prior to and following surgical intervention.

## Conclusions

GATT ab-interno is a major advancement in PCG management as a minimally invasive glaucoma surgery, as it does not injure conjunctival or scleral tissue and, therefore, should not interfere with future filtration or drainage implant surgery. However, it necessitates expertise in angle anatomy, gonioscopy, and meticulous surgical skills to reduce complications and enhance postoperative results. As long as the presenting IOP is not too high and the disc damage is mild to moderate, the hemi-GATT can effectively reduce IOP in patients with PCG, for which we need additional studies with long-term follow-up to establish it.
